# The contribution of databases to the results of systematic reviews: a cross-sectional study

**DOI:** 10.1186/s12874-016-0232-1

**Published:** 2016-09-26

**Authors:** Lisa Hartling, Robin Featherstone, Megan Nuspl, Kassi Shave, Donna M. Dryden, Ben Vandermeer

**Affiliations:** 1 Cochrane Child Health, University of Alberta, ECHA4-472, 11405-87 Avenue, Edmonton, AB T6G 1C9 Canada; 2 Alberta Research Center for Health Evidence (ARCHE), University of Alberta, 11405-87 Avenue, Edmonton, AB T6G 1C9 Canada

**Keywords:** Systematic reviews, Literature searching, Meta-analysis, Knowledge synthesis, Bias

## Abstract

**Background:**

One of the best sources for high quality information about healthcare interventions is a systematic review. A well-conducted systematic review includes a comprehensive literature search. There is limited empiric evidence to guide the extent of searching, in particular the number of electronic databases that should be searched. We conducted a cross-sectional quantitative analysis to examine the potential impact of selective database searching on results of meta-analyses.

**Methods:**

Our sample included systematic reviews (SRs) with at least one meta-analysis from three Cochrane Review Groups: Acute Respiratory Infections (ARI), Infectious Diseases (ID), Developmental Psychosocial and Learning Problems (DPLP) (*n* = 129). Outcomes included: 1) proportion of relevant studies indexed in each of 10 databases; and 2) changes in results and statistical significance of primary meta-analysis for studies identified in Medline only and in Medline plus each of the other databases.

**Results:**

Due to variation across topics, we present results by group (ARI *n* = 57, ID *n* = 38, DPLP *n* = 34). For ARI, identification of relevant studies was highest for Medline (85 %) and Embase (80 %). Restricting meta-analyses to trials that appeared in Medline + Embase yielded fewest changes in statistical significance: 53/55 meta-analyses showed no change. Point estimates changed in 12 cases; in 7 the change was less than 20 %. For ID, yield was highest for Medline (92 %), Embase (81 %), and BIOSIS (67 %). Restricting meta-analyses to trials that appeared in Medline + BIOSIS yielded fewest changes with 1 meta-analysis changing in statistical significance. Point estimates changed in 8 of 31 meta-analyses; change less than 20 % in all cases. For DPLP, identification of relevant studies was highest for Medline (75 %) and Embase (62 %). Restricting meta-analyses to trials that appeared in Medline + PsycINFO resulted in only one change in significance. Point estimates changed for 13 of 33 meta-analyses; less than 20 % in 9 cases.

**Conclusions:**

Majority of relevant studies can be found within a limited number of databases. Results of meta-analyses based on the majority of studies did not differ in most cases. There were very few cases of changes in statistical significance. Effect estimates changed in a minority of meta-analyses but in most the change was small. Results did not change in a systematic manner (i.e., regularly over- or underestimating treatment effects), suggesting that selective searching may not introduce bias in terms of effect estimates.

**Electronic supplementary material:**

The online version of this article (doi:10.1186/s12874-016-0232-1) contains supplementary material, which is available to authorized users.

## Background

The realization of effective and efficient health care services requires that decisions are informed by the best available evidence. Arguably the best source for such information is high quality knowledge syntheses, such as systematic reviews (SRs). One of the hallmarks of a well-conducted SR is a thorough, objective and reproducible search of a range of sources to identify as many relevant studies as possible, to minimize bias and assist in achieving reliable estimates of effects [1]. Details on the extent of the search, and in particular the number of electronic databases that should be searched, however, are not available. The Cochrane Handbook states that the search should be as extensive as possible and that the Cochrane Central Register of Controlled Trials (CENTRAL), Medline and Embase are the most important sources to search for studies for inclusion in Cochrane reviews [1]. Methodological standards for the conduct of new Cochrane interventions require searches of CENTRAL, Medline and Embase, and reviewers are encouraged to consider subject specific databases (e.g. CINAHL for nursing related topics, or PsycINFO for psychological interventions) and regional databases (e.g. LILACS) [2]. Guidance on the contributions of international, national, regional and/or subject specific databases, however, is not specific. Standards for SRs from the Institute of Medicine acknowledge that “little empirical evidence is available to guide the development of an SR bibliographic search strategy” ([3], p74). The standards list several bibliographic databases including CENTRAL, the Database of Abstracts of Reviews of Effectiveness (DARE), Embase, Medline, as well as two regional databases from Africa and the Caribbean; however, the recommendations are not explicit about which and how many databases must be searched.

Much of the empirical evidence that exists for questions of searching involves examining the sensitivity and precision of different databases and search filters in terms of study identification [4–22]. One important gap is the modest amount of empirical evidence demonstrating the impact on results and conclusions from different approaches to searching. In 2003, Sampson et al. published a paper investigating the impact of including trials indexed only in Embase on the effect estimate of a meta-analysis [23]. Among 968 randomized controlled trials from 61 meta-analyses, 10 % were found in Embase but not Medline. In 24 meta-analyses, including Embase-unique trials decreased interventions effects by 8 %. The authors recommended further investigation in recognition of ongoing changes to bibliographic databases. In an analysis of 44 systematic reviews on diabetes interventions, it was shown that basing a meta-analysis only on the results of a Medline search would miss trials that could affect the result 34 % of the time [24]. In 2015, Halladay et al. investigated the impact of using sources beyond PubMed (Medline) in systematic reviews. The authors randomly selected 50 Cochrane Reviews that searched PubMed and Embase and included a meta-analysis of ≥10 studies. They examined if excluding the studies not found in PubMed affected results, and found that meta-analysis using only PubMed-indexed versus all available studies led to a different conclusion in only a single case [25].

The evidence-base on database selection for systematic reviews is limited to showing the impact of searching Medline only, or excluding Embase. To our knowledge, the contribution of a range of databases or the combination of different database selections on the results of meta-analysis has not been reported. Further, advances over recent years, including the increased scope of Medline and Embase, and allied health databases (e.g., CINAHL, PsycINFO), registration of clinical studies, and the increasing reliance on web-based searching to locate grey literature, raise questions about indicators of comprehensive literature searches. While methodological guidance for SRs encourages comprehensive searching, there are diminishing returns with each additional database searched [7] and the impact of searching each additional database in terms of the final results and conclusions is not known. Moreover, more serendipitous discovery methods (e.g., cited reference searching) may yield more relevant studies than more database searching [6, 10, 13, 19]. In practice, reasonable limits must be placed on the number of sources to be searched, and currently these limits are determined in the absence of any broad agreement around stopping rules for searching. The objective of this study was to examine the potential impact on the results of existing SRs if searching was restricted to select bibliographic databases.

## Methods

### Sample

The sample was derived from a register of SRs that we maintain as part of our mandate with Cochrane Child Health. The register includes approximately 1,400 SRs relevant to child health that are published in the Cochrane Database of Systematic Reviews (CDSR). The CDSR was chosen for this analysis as Cochrane reviews: 1) provide tabulated data from the component trials allowing for re-analysis; 2) provide a detailed list of references for all relevant trials; and, 3) have been reported to be of higher methodological quality [26, 27], which may translate into more comprehensive searches, hence more ability to examine our hypothesis around extent of searching and impact on results and conclusions. As part of earlier work, we extracted information from each of the SRs including detailed information on the search strategy (e.g., databases searched) and the data used from the individual studies for the primary analysis [28]. The register has been updated on a regular basis as part of the ongoing research within Cochrane Child Health. For this project, we included all reviews containing at least one meta-analysis from the three review groups contributing the most reviews: Acute Respiratory Infections (ARI; *n* = 57), Infectious Diseases (ID; *n* = 38), and Development, Psychosocial and Learning Problems (DPLP; *n* = 34). We included reviews regardless of the nature of the outcome, i.e., we included meta-analyses of dichotomous and continuous outcomes.

### Analysis

To determine our set of databases for investigation we sampled 50 reviews conducted by three Cochrane review groups (ARI, DPLP and Airways) and developed a preliminary list of 108 information sources. From the preliminary list, we excluded meta-search databases (e.g. SciSearch), citation databases (e.g. Scopus, Web of Science), trial registry databases (e.g. metaRegister of Controlled Trials), regional subsets of Medline (e.g. African Index Medicus), dissertation databases (e.g. Australasian Digital Theses Program), and highly specialized databases (e.g. Bibliography of Nordic Criminology, European Committee for Homeopathy thesis database). We excluded citation databases (Scopus, Web of Science) as the selected studies from our sample set of Cochrane reviews may have been added to these databases only after the reviews were completed and as a result of being referenced in them. CENTRAL was also excluded since all included trials in published Cochrane reviews are added to CENTRAL. Including CENTRAL, or any citation databases, would bias our results in favour of these sources, and we could not assume included studies would have been located in these sources prior to the publication of the Cochrane reviews in our sample.

The remaining information sources were reviewed by our research librarian (RF) and a second author (DMD). We selected the ten databases as those most likely to be searched in SRs of healthcare interventions: Ovid Medline (1946-Current), BIOSIS Citation Index via Thomson Reuters Web of Knowledge (1926-Current), CAB Direct via CABI (1910-Current), CINAHL Plus with Full Text via EBSCOhost (1937-Current), Ovid Embase (1974-Current), Ovid ERIC (1965-Current), Ovid HaPI (Health and Psychosocial Instruments) (1985-Current), Ovid IPA (International Pharmaceutical Abstracts) (1965-Current), LILACS via BIREME Virtual health Library (inception-Current), and Ovid PsycINFO (1806-Current). Given the high overlap between Ovid Medline and the PubMed interface via NCBI Entrez, and the frequent use of Ovid Medline for Cochrane reviews, we selected the Ovid interface for searching studies included in Medline. Similarly, we selected Ovid Embase over Embase.com, in this case as only the Ovid version of the database was available at our center.

We then listed all of the studies included in the primary meta-analysis for each SR (i.e., our reference standard). We chose the meta-analysis that was designated as the primary outcome by the authors or, if not specified, we selected the first meta-analysis presented in the review. We assumed the first meta-analysis presented would be one of the more important outcomes. This also typically contained the most studies, providing more data to run our analyses. Our analysis involved five key components:

1. For each meta-analysis, we searched the ten databases listed above to determine the number of trials contained in each. We calculated the mean percentage of trials per meta-analysis contained in each database, as well as the minimum, first quartile, median, third quartile, and maximum.
2. We recorded how many of the studies not indexed in Medline were indexed in each of the additional databases. For the DPLP reviews, we found a relatively lower proportion of studies indexed in Medline and larger proportion in PsycINFO; therefore, we based our analyses on studies found (or not found) in Medline and/or PsycINFO.
3. For each meta-analysis, we re-analyzed the data based on studies that were identified only in Medline and Medline plus each of the additional databases (e.g., Medline + BIOSIS, Medline + Embase, etc.). For DPLP reviews we conducted this analysis for Medline + PsycINFO and then Medline + PsycINFO along with each additional database. For dichotomous outcomes, we calculated the mean of the ratios of point estimates for Medline and Medline + additional database relative to the original meta-analysis (i.e., reference standard). We calculated the mean confidence interval widths on the log scale of the ratios of confidence interval widths for Medline and Medline + additional database relative to the reference standard. For continuous outcomes, we calculated the mean of the standardized differences of effect sizes and mean ratios of confidence interval widths. For all analyses, we also calculated the minimum, first quartile, median, third quartile, and maximum ratios. When computing ratios of point estimates and confidence intervals, we ignored the direction of effect and considered only the magnitude; that is to say for each meta-analysis, if the ratio was less than 1, we replaced it with its reciprocal. Thus the minimum value of any ratio was 1. In this way we would see the magnitude of the differences rather than allowing over- and under-estimates to potentially cancel each other by giving us a mean ratio that was close to 1.
4. We recorded the number of times the statistical significance of the result changed and categorized changes as: reference standard significant and Medline or Medline plus additional database not significant, or reference standard not significant and Medline or Medline plus additional database significant.
5. Finally, we examined in more detail the meta-analyses that had changes in effects based on selective database searching to understand the frequency and extent of changes, and whether there were patterns relative to the number of relevant studies and proportion retrieved through selective searching.

Statistical analyses were conducted using SAS 9.3 (SAS institute Inc., Cary, NC USA). Results are presented by Review Group (i.e., ARI, ID, DPLP) due to heterogeneity in some findings across groups.

## Results

Our analyses are based on 57 meta-analyses from ARI, 38 from ID, and 34 from DPLP (See Additional file 1). The median years of publication for the SRs used in our analysis were 2012 (ARI), 2008.5 (ID), and 2011 (DPLP). The median numbers of studies (and participants) included in the primary or first meta-analysis were 4 (1,031) for ARI, 3 (553) for ID, and 3 (308 for DPLP).

Table 1 shows the mean percent of trials from the original meta-analyses that were found in each database. For ARI, a mean of 85 % of trials was identified in Medline (median 100 %), followed by Embase (80 %, median 100 %), and BIOSIS (65 %, median 67 %). The pattern was similar for ID, with a mean of 92 % of trials identified in Medline (median 100 %), followed by Embase (81 %, median 86 %), and BIOSIS (67 %, median 68 %). Likewise, for DPLP a mean of 75 % was identified in Medline (median 100 %), followed by Embase (62 %, median 75 %), and BIOSIS (49 %, median 100 %).

**Table 1:** Mean percent of trials from original meta-analysis found in each database^a^

Database	Mean %	Minimum %	Q1 %	Median %	Q3 %	Maximum %
Acute Respiratory Infections (*n* = 57)						
Medline	85	0	79	100	100	100
BIOSIS	65	0	50	67	100	100
CAB Direct	0.3	0	0	0	0	20
CINAHL	22	0	0	0	33	100
EMBASE	80	0	75	100	100	100
ERIC	0.2	0	0	0	0	13
HAPI	0	0	0	0	0	0
IPA	23	0	0	20	42	100
LILACS	0	0	0	0	0	0
PsycINFO	2	0	0	0	0	88
Infectious Diseases (*n* = 38)						
Medline	92	33	86	100	100	100
BIOSIS	67	0	50	68	100	100
CAB Direct	5	0	0	0	0	100
CINAHL	10	0	0	0	17	50
EMBASE	81	20	67	86	100	100
ERIC	0	0	0	0	0	0
HAPI	0	0	0	0	0	0
IPA	17	0	0	1	29	100
LILACS	5	0	0	0	0	100
PsycINFO	3	0	0	0	0	100
Developmental Psychosocial Learning Problems (*n* = 34)						
Medline	75	0	50	100	100	100
BIOSIS	49	0	0	50	88	100
CAB Direct	0	0	0	0	0	0
CINAHL	34	0	0	35	50	100
EMBASE	62	0	25	75	100	100
ERIC	26	0	0	0	50	100
HAPI	0	0	0	0	0	0
IPA	7	0	0	0	0	67
LILACS	3	0	0	0	0	100
PsycINFO	47	0	0	50	90	100

^a^Percentages do not match those in Table 2; the percentages here are an average across meta-analyses

Table 2 shows the databases where trials were found that were not found in Medline. Eighty-four percent of all trials contained in ARI meta-analyses (333/398) were found in Medline. Among the remaining 65 trials, 20 were found in Embase and 13 were found in BIOSIS. The remaining databases found very few trials that were not identified in Medline. Eighty-seven percent of all trials contained in ID meta-analyses (206/238) were found in Medline. Among the remaining 32 trials, 7 were found in BIOSIS and 6 were found in Embase; the remaining databases only contained 0 or 1 each. Due to the relatively lower proportion of trials found in Medline alone for DPLP, we combined Medline and PsycINFO which together contained 84 % of trials (121/144). Of the remaining 23, 4 were found in ERIC, 2 in Embase and 1 in BIOSIS; the remaining databases contained 0.

**Table 2:** Databases where trials were found that were not found in Medline^a^

	Acute Respiratory Infections (*n* = 57 meta-analyses)		Infectious Diseases (*n* = 38 meta-analyses)		Developmental, Psychosocial and Learning Problems (*n* = 34 meta-analyses)	
Database	Total Trials Found (of 398)^b^	Number found that were not in MedLine (of 65)	Total Trials Found (of 238)^b^	Number found that were not in MedLine (of 32)	Total Trials Found (of 144)^b^	Number found that were not in MedLine or PsycINFO (of 23)
Medline	333 (84 %)	-	206 (87 %)	-	-	-
BIOSIS	258	13	164	7	72	1
CAB Direct	1	0	4	0	0	0
CINAHL	75	0	32	1	43	0
EMBASE	310	20	178	6	84	2
ERIC	1	1	0	0	32	4
HAPI	0	0	0	0	0	0
IPA	104	3	40	1	13	0
LILACS	0	0	5	0	2	0
PsycINFO	8	3	2	0	121 (84 %)^c^	-

^a^Percentages do not match those in Table 1; the percentages here are an average across all trials (by review group)

^b^Number of trials not found in any database was 26 for ARI, 18 for ID, and 12 for DPLP. Number of systematic reviews with ≥1 trial not found in any database was 10 for ARI, 9 for ID, and 8 for DPLP

^c^Total trials found in Medline and/or PsycINFO

In terms of the impact on the statistical significance of results (Table 3), Medline + Embase yielded the fewest changes for ARI with 53 of the 55 meta-analyses showing no change, while one meta-analysis changed from significant to non-significant and one meta-analysis changed from non-significant to significant. For ID, Medline + BIOSIS yielded the fewest changes with only 1 meta-analysis changing from significant to non-significant. All other analyses (Medline alone and Medline + each additional database) resulted in only 2 changes (one in each direction). For DPLP, all combinations (Medline + PsycINFO and Medline + PsycINFO + additional database) resulted in only one change (significant to non-significant).

**Table 3:** Impact of selective searching on statistical significance of results

	Acute Respiratory Infections (*n* = 55)			Infectious Diseases (*n* = 37)			Developmental, Psychosocial and Learning Problems (*n* = 33)		
Database^a^	Reference significant; Medline+ not	Reference not significant; Medline+ significant	No change	Reference significant; Medline+ not	Reference not significant; Medline+ significant	No change	Reference significant; Medline+ not	Reference not significant; Medline+ significant	No change
Medline (alone)	4	2	49	1	1	35	-	-	-
BIOSIS	3	2	50	0	1	36	1	0	32
CAB Direct	4	2	49	1	1	35	1	0	32
CINAHL	4	2	49	1	1	35	1	0	32
EMBASE	1	1	53	1	1	35	1	0	32
ERIC	3	2	50	1	1	35	1	0	32
HAPI	4	2	49	1	1	35	1	0	32
IPA	4	2	49	1	1	35	1	0	32
LILACS	4	2	49	1	1	35	1	0	32
PsycINFO	3	2	50	1	1	35	1	0	32

^a^each database in addition to Medline

We calculated the mean of the ratios and confidence interval widths for point estimates of Medline alone and Medline + each database relative to the reference standard for dichotomous outcomes. For ARI and ID, the lowest mean ratios were found for Medline + BIOSIS (1.03 ARI, 1.02 ID) and Medline + Embase (1.05 ARI, 1.02 ID), although the ratios were low in all cases. The ratios of the confidence interval widths were consistent with the ratios of the point estimate. For DPLP, all ratios were the same at 1.04 for Medline + PsycINFO and Medline + PsycINFO with each additional database. We conducted similar analyses based on standardized differences of effect sizes for continuous outcomes. The mean of the standardized differences of point estimates was 0.01 for ARI reviews for all analyses except one (Medline + IPA) and the mean of the ratios of confidence interval widths differed little across cases, ranging from 1.15 to 1.17. For ID, the mean of the standardized differences of point estimates showed little variation, ranging from 0.0004 to 0.0006, with the mean of the ratios of confidence interval widths ranging from 1.26 to 1.28. For DPLP, the mean of the standardized differences of point estimates was 0.01 for all comparisons except Medline + PsycINFO + ERIC which was 0.004. The mean of the ratios of the confidence interval widths ranged from 1.09 to 1.11. Detailed results from these analyses are available in the Appendix.

We examined the specific meta-analyses in more detail to contextualize the above results. For ARI, the results of only 17 of the 57 meta-analyses changed when restricting included studies to those found in Medline + BIOSIS (12 of 57 for Medline + Embase; Table 4). Therefore, the above results demonstrating changes to point estimates and confidence intervals are driven by a minority of the meta-analyses and cannot necessarily be generalized (or averaged) to any specific meta-analysis. Among the 17 meta-analyses, the proportion of relevant studies identified by Medline + BIOSIS ranged from 0 to 93 % (median 66 %). Typically when a low proportion was identified, the original meta-analyses had included relatively few studies (i.e., in all cases where less than 50 % of relevant studies were found in Medline + BIOSIS, the original meta-analysis had 6 or fewer studies). The point estimate changed less than 20 % in most cases (*n* = 12 of 17). In two cases no studies were found in Medline + BIOSIS; in both cases the original meta-analysis only included 2 studies and both involved complementary medicine interventions for influenza (i.e., homeopathic oscillococcinum and Chinese medicinal herbs).[29, 30] The 12 meta-analyses that changed when restricting studies to those found in Medline + Embase were a subset of the 17 analyzed for Medline + BIOSIS and followed the same patterns (see Table 4).

**Table 4:** Impact of selective searching on results of meta-analyses from acute respiratory infections

		Original meta-analysis				Meta-analysis based on studies identified by selective search					
Case	Summary measure	Number of trials	Effect size	Lower CI	Upper CI	Number of trials	Effect size	Lower CI	Upper CI	% of trials identified	% change in effect size
Medline + BIOSIS (changes in 17 of 57 meta-analyses)											
1^a^	MD	5	−21.29	−29.59	−13	1	−22.9	−41.34	−4.46	20	7.0
2^a^	MD	35	−1.24	−1.54	−0.94	26	−1.42	−1.77	−1.06	74	12.7
3^a^	MD	24	−0.45	−0.96	0.05	19	−0.25	−0.9	0.39	79	44.7
4^a^	RD	2	−0.13	−0.21	−0.05	1	−0.16	−0.26	−0.05	50	18.8
5^a^	RD	3	−0.05	−0.12	0.02	1	−0.04	−0.14	0.06	33	20.0
6	MH OR	6	0.27	0.15	0.50	3	0.19	0.08	0.46	50	29.6
7^a, b^	MH OR	7	0.35	0.12	1.01	6	0.26	0.12	0.6	86	25.7
8^a^	RR	2	0.48	0.17	1.34	0	-	-	-	0	-
9^a, b^	SMD	8	0.49	0.16	0.81	6	0.51	0.1	0.93	75	3.9
10	MD	3	0.5	0.10	0.90	2	0.43	−0.01	0.87	67	14.0
11	RR	6	0.67	0.50	0.89	5	0.75	0.55	1.04	83	10.7
12	RR	25	0.9	0.80	1.01	22	0.91	0.8	1.02	88	1.1
13^a^	RR	6	0.95	0.59	1.51	3	0.96	0.82	1.11	50	1.0
14^a, b^	RR	29	0.95	0.92	0.98	19	0.96	0.94	0.99	66	1.0
15^a, b^	RR	15	1.09	0.64	1.85	13	0.9	0.54	1.49	87	17.4
16^a^	RR	2	1.32	0.87	2.00	0	-	-	-	0	-
17	RR	15	1.82	0.98	3.38	14	1.97	1.06	3.64	93	7.6
Medline + Embase (changes in 12 of 57 meta-analyses; data below show cases where results were different from Medline + BIOSIS results above^b^)											
7	MH OR	7	0.35	0.12	1.01	4	0.14	0.04	0.53	57	60.0
9	SMD	8	0.49	0.16	0.81	6	0.4	0.07	0.73	75	18.4
14	RR	29	0.95	0.92	0.98	19	0.97	0.94	1	66	2.1
15	RR	15	1.09	0.64	1.85	14	0.96	0.58	1.57	93	11.9

^a^cases where results also changed for Medline + Embase; ^b^cases where results were different between Medline + BIOSIS and Medline + Embase analyses

*CI* confidence interval, *MD* mean difference, *MH OR* Mantel Haenszel odds ratio, *RD* risk difference, *RR* risk ratio, *SMD* standardized mean difference

For ID, the results of only 9 of 31 meta-analyses changed when restricting included studies to those found in Medline + Embase (8 of 31 for Medline + BIOSIS; Table 5). Among the 9 meta-analyses, the proportion of relevant studies identified by Medline + Embase ranged from 60 to 86 % (median 75 %). As for ARI, when a lower proportion was identified, the original meta-analyses had included relatively few studies. The point estimate changed less than 20 % in all cases and less than 10 % in 7 of 9 cases. The 8 meta-analyses that changed when restricting studies to those found in Medline + BIOSIS were a subset of the 9 analyzed for Medline + Embase and are presented in Table 5.

**Table 5:** Impact of selective searching on results of meta-analysis from infectious diseases

		Original meta-analysis				Meta-analysis based on studies identified by selective search					
Case	Summary measure	Number of trials	Effect size	Lower CI	Upper CI	Number of trials	Effect size	Lower CI	Upper CI	% of trials identified	% change in effect size
Medline + Embase (changes in 9 of 31 meta-analyses)											
1^a, b^	MD	35	−24.76	−33.61	−15.91	30	−22.22	−31.85	−12.6	86	10.3
2^a, b^	RR	5	0.41	0.11	1.64	3	NE	-	-	60	-
3	RR	4	0.48	0.35	0.65	3	0.57	0.29	1.09	75	15.8
4^a, b^	RR	21	0.53	0.49	0.57	16	0.51	0.46	0.56	76	3.8
5^a, b^	MH OR	11	0.59	0.45	0.79	7	0.61	0.41	0.89	64	3.3
6^a^	RR	3	0.62	0.19	2.04	2	0.56	0.14	2.14	67	9.7
7^a, b^	MH OR	10	0.68	0.49	0.95	8	0.59	0.41	0.86	80	13.2
8^a^	RR	8	0.71	0.62	0.80	6	0.72	0.64	0.82	75	1.4
9^a^	RR	13	0.99	0.90	1.09	11	0.95	0.86	1.05	85	4.0
Medline + BIOSIS (changes in 8 of 31 meta-analyses; data below show cases where results were different from Medline + Embase results above^b^)											
1	MD	35	−24.76	−33.61	−15.91	29	−23.18	−32.94	−13.42	83	6.4
2	RR	5	0.41	0.11	1.64	3	NE	-	-	60	-
4	RR	21	0.53	0.49	0.57	17	0.49	0.44	0.54	81	7.5
5	MH OR	11	0.59	0.45	0.79	8	0.64	0.48	0.87	73	7.8
7	MH OR	10	0.68	0.49	0.95	8	0.54	0.34	0.86	80	20.6

*CI* confidence interval, *MD* mean difference, *MH OR* Mantel Haenszel odds ratio, *NE* not estimable (all study groups had zero counts); *RR* risk ratio

^a^cases where results also changed for Medline + BIOSIS; ^b^cases where results were different between Medline + BIOSIS and Medline + Embase analyses

For DPLP, there were 15 of 33 meta-analyses that changed when restricting included studies to those found in Medline + PsycInfo (13 of 33 for Medline + PsycInfo + ERIC; Table 6). Among the 15 meta-analyses, the proportion of relevant studies identified in Medline + PsycInfo ranged from 0 to 94 % (median 75 %). As for ARI and ID, when a lower proportion was identified, the original meta-analyses had included relatively few studies. For example, in the case where there were no studies found in Medline + PsycInfo, the original meta-analysis only had 2 studies and was on a topic not traditionally considered as a healthcare intervention (i.e., restorative justice conferencing for reducing recidivism in young offenders) [31]. In two cases the original meta-analyses had 2 included studies and Medline + PsycInfo only identified 1 (50 %); topics were music therapy for autism spectrum disorder and financial benefits for child health and well-being in low income or socially disadvantaged families in developed world countries [32, 33]. The point estimate changed less than 20 % in 13 cases. Data for Medline + PsycInfo + ERIC are presented in Table 6.

**Table 6:** Impact of selective searching on results of meta-analysis from developmental, psychosocial and learning problems

		Original meta-analysis				Meta-analysis based on studies identified by selective search					
Case	Summary measure	Number of trials	Effect size	Lower CI	Upper CI	Number of trials	Effect size	Lower CI	Upper CI	% of trials identified	% change in effect size
Medline + PsycInfo (changes in 15 of 33 meta-analyses)											
1^a, b^	MD	5	−1.25	−4.56	2.06	3	−1.48	−5.96	3	60	15.5
2^a^	SMD	10	−0.24	−0.35	−0.13	8	−0.25	−0.38	−0.11	80	4.0
3^a^	MD	5	0.02	−0.09	0.12	4	0	−0.11	0.11	80	NA
4	SMD	10	0.47	0.06	0.88	9	0.49	0.04	0.93	90	4.1
5^a^	SMD	2	0.5	0.22	0.79	1	0.48	0.18	0.77	50	4.0
6^a^	RR	10	0.51	0.37	0.72	8	0.48	0.34	0.69	80	5.9
7^a^	MH OR	4	0.61	0.27	1.39	3	0.48	0.14	1.6	75	21.3
8^a^	RR	6	0.69	0.60	0.78	5	0.66	0.59	0.74	83	4.3
9^a, b^	RR	10	0.73	0.56	0.95	7	0.65	0.47	0.9	70	11.0
10^a^	RR	17	0.76	0.69	0.83	16	0.75	0.68	0.82	94	1.3
11^a^	MH OR	4	0.87	0.61	1.25	3	0.78	0.52	1.17	75	10.3
12^a^	Other	2	1	0.59	1.71	0	-	-	-	0	-
13^a, b^	MH OR	2	1.05	0.90	1.23	1	1.09	0.85	1.41	50	3.7
14^a^	MH OR	7	1.68	1.20	2.36	2	1.78	0.93	3.41	29	5.6
15	MD	4	14.37	12.30	16.44	3	12.33	10.14	14.51	75	14.2
Medline + PsycInfo + ERIC (changes in 13 of 33 meta-analyses; data below show cases where results were different from Medline + PsycInfo results above^b^)											
1	MD	5	−1.25	−4.56	2.06	4	−0.96	−5.07	3.15	0.8	23.2
9	RR	10	0.73	0.56	0.95	9	0.69	0.52	0.92	0.9	5.5
13	MH OR	2	1.05	0.90	1.23	2	1.09	0.91	1.31	1	3.7

*CI* confidence interval, *MD* mean difference, *MH OR* Mantel Haenszel odds ratio, *NA* not applicable, *RR* risk ratio, *SMD* standardized mean difference

^a^cases where results also changed for Medline + PsycInfo + ERIC; ^b^cases where results were different between Medline + PsycInfo and Medline + PsycInfo + ERIC

## Discussion

Systematic reviews are critical for informed, evidence-based decision-making. They have been described as the cornerstone of knowledge translation and are the foundation for key knowledge tools such as clinical practice guidelines and patient decision aids [34]. One of the fundamental components and initial steps in conducting a SR is the literature search. Best practices recommend extensive searching to ensure comprehensive identification of all studies relevant to the question of interest and to avoid bias in results and conclusions. However, extensive searching contributes substantially to the workload, resources, and time required to complete a SR. Moreover, there is limited empiric evidence upon which to base the extent of searching or the potential impact of selective searching on the results of SRs.

This study provides much needed empiric evidence to support prioritizing particular databases to search in SRs. Our results show that the vast majority of relevant studies appear within a limited number of databases. Further, the results of meta-analyses based on the majority of studies (that appear within a limited number of databases) do not differ in the majority of cases. In particular, there were very few cases of results changing in statistical significance. The effect estimates changed in a minority of meta-analyses but in the majority of these they changed to a small extent. Finally, results do not appear to change in a systematic manner (i.e., regularly over- or underestimating treatment effects), suggesting that searching select databases may not introduce bias in terms of effect estimates.

While our results suggest that the majority of relevant studies appear in a limited number of databases, the choice of databases is topic-specific. We purposefully selected three different clinical areas to examine our hypothesis. For two of the clinical areas (acute respiratory infections and infectious diseases), we found the highest yield from Medline + Embase or Medline + BIOSIS. Further, these combinations resulted in the least impact on effect estimates and fewest changes in statistical significance of results. For the third area (Developmental, Psychosocial and Learning Problems), Medline + PsycInfo yielded the most relevant studies, with ERIC contributing more additional studies than other databases including Embase or BIOSIS. Further research in these and other topic areas is needed to provide empiric evidence to inform searching and optimize the time and resources that are required to produce SRs. In cases where we found a very low proportion of studies in select databases, the original meta-analyses included few studies and topics were often outside of mainstream healthcare interventions (e.g., complementary and alternative medicine, financial benefits, restorative justice). Further, our results are based on SRs that focused on randomized trials; these results may not be generalizable to other study designs or types of data.

For those conducting SRs, it may be appropriate to limit the number of databases being searched for mainstream healthcare interventions. If reviewers anticipate a small number of studies (based on preliminary searches conducted as part of developing the SR protocol and the complete search strategy), they may choose to search a larger number of databases at the outset; if they find a small number of studies having searched few databases, they may choose to search more (e.g., an iterative approach), or supplement with other sources such as those suggested for identification of grey literature (e.g., content experts, relevant websites, conferences, etc.). Our results may be particularly pertinent in the context of rapid reviews. There has been increased attention recently to methods for rapid reviews in the interests of producing knowledge syntheses more quickly and efficiently to inform end-users’ decision-making needs [35–37]. Searching is one dimension of SRs that is typically altered to streamline processes and produce reviews more quickly [37, 38]. Alterations include limiting the number of databases and extent of grey literature searching, and placing restrictions on date, setting, language and study design. Further, end-users of SRs have indicated that extent of literature searching is one of the most acceptable trade-offs to increase review efficiencies [39].

Our study had several limitations. First, we focused on a sample of SRs published in the Cochrane Database of Systematic Reviews. These were from three clinical areas and focused on healthcare interventions. Results may not be generalizable outside of these clinical areas, for non-conventional interventions, or for SRs examining other types of research questions (e.g., diagnostic, prognostic). Second, our sample was based on SRs that had already been completed; we used the original search strategies and the studies that they yielded as our reference standard. There may have been variability in the comprehensiveness of the original search strategies; however, Cochrane reviews are recognized as high quality (including criterion related to searching) and typically search specialized registers that include studies identified through extensive searching activities including hand-searching. Third, we did not evaluate the searches per se, rather we looked to see whether the included studies could be found in each database. Therefore, our results are based on whether the studies were present in the databases but do not reflect the ability of searchers (or a given search strategy) to find those references. Further, since our study was retrospective, some of the studies may have been deposited into the databases after the original search was run; therefore, our results may overestimate the identification of studies that are published and indexed closer to the time that the search is implemented. Fourth, we focused on analyses on the primary (or first listed) outcome from each review. Results may vary across outcomes; however, our focus on the primary outcome provided the most data with which to examine our hypotheses. Fifth, to avoid biasing our results in favor of sources which included studies referenced in our sample set of Cochrane reviews, we were not able to evaluate the relative contributions from searching CENTRAL and citation databases (Web of Science and Scopus). Sixth, we did not include the PubMed interface via NCBI Entrez for Medline or Embase.com for Embase (https://www.nlm.nih.gov/pubs/factsheets/dif_med_pub.html). There may be studies located in PubMed or Embase.com that may not have been found in Ovid Medline or Ovid Embase [40]. Finally, we only examined the impact of Medline and Medline plus one other database. Further research examining the contributions of different numbers of databases, ranked according to their potential for identifying relevant studies in a specific topic area, may be beneficial.

This study confirms previous methods studies demonstrating that the majority of SR trials are found in Medline [7, 25], and that Embase is more likely than other databases to find additional relevant trials not retrieved by Medline [23, 24]. Our study contributes evidence to support the addition of subject-specific database (PsycINFO, ERIC) for specific topics, and the contribution of Biosis in addition to Embase for clinical medicine topics. While this study provides important information, future research in this area is needed to help guide searching in SRs, and to quantify the impact of different approaches to searching on the results and conclusions of reviews. As mentioned above, our results reflect the presence of studies in the different databases but do not reflect the ability of searches to identify those studies. Research evaluating selective searching in a prospective manner and in different topic areas would be beneficial. Moreover, additional empiric research will help provide more solid evidence upon which to base recommendations for searching in the future.

## Conclusions

This study provides quantitative data regarding the potential impact on meta-analysis results of restricting searches to select databases. The vast majority of relevant studies appear within a limited number of databases. The results of meta-analyses based on the majority of studies (which appear within a limited number of databases) did not differ in most cases; specifically, there were very few cases of results changing in statistical significance. Effect estimates changed in a minority of meta-analyses but in most the change was small. Results did not change in a systematic manner (i.e., regularly over- or underestimating treatment effects), suggesting that selective searching may not introduce bias in terms of effect estimates. This information may be useful to increase efficiencies in the conduct of SRs and in developing methods guidance for rapid reviews. Future research across different topics will provide additional evidence upon which to base recommendations for searching in evidence reviews.

## Appendix

Detailed results data

This file includes four tables with the following data: 1) mean of the ratios of point estimate of Medline/database estimate to reference standard point estimate for dichotomous outcomes, 2) mean (on the log scale) of the ratios of confidence interval widths of Medline/database estimate to reference standard point estimate for dichotomous outcomes, 3) mean of standardized differences of effect sizes for continuous outcomes, 4) mean of the ratios of confidence interval widths of Medline/database estimate to reference standard point estimate for dichotomous outcomes.

**Table 7:** Mean of the ratios of point estimate of Medline/database estimate to reference standard point estimate for dichotomous outcomes

Database	Mean	Minimum	Q1	Median	Q3	Maximum
Acute Respiratory Infections (*n* = 37)						
Medline (alone)	1.06	1.00	1.00	1.00	1.00	2.5
BIOSIS	1.03	1.00	1.00	1.00	1.00	1.4
CAB Direct	1.06	1.00	1.00	1.00	1.00	2.5
CINAHL	1.06	1.00	1.00	1.00	1.00	2.5
EMBASE	1.05	1.00	1.00	1.00	1.00	2.5
ERIC	1.06	1.00	1.00	1.00	1.00	2.5
HAPI	1.06	1.00	1.00	1.00	1.00	2.5
IPA	1.07	1.00	1.00	1.00	1.00	2.5
LILACS	1.06	1.00	1.00	1.00	1.00	2.5
PsycINFO	1.06	1.00	1.00	1.00	1.00	2.5
Infectious Diseases (*n* = 31)						
Medline (alone)	1.06	1.00	1.00	1.00	1.00	2.14
BIOSIS	1.02	1.00	1.00	1.00	1.00	1.26
CAB Direct	1.06	1.00	1.00	1.00	1.00	2.14
CINAHL	1.06	1.00	1.00	1.00	1.00	2.14
EMBASE	1.02	1.00	1.00	1.00	1.00	1.19
ERIC	1.06	1.00	1.00	1.00	1.00	2.14
HAPI	1.06	1.00	1.00	1.00	1.00	2.14
IPA	1.05	1.00	1.00	1.00	1.00	2.14
LILACS	1.06	1.00	1.00	1.00	1.00	2.14
PsycINFO	1.06	1.00	1.00	1.00	1.00	2.14
Developmental, Psychosocial and Learning Problems (*n* = 16)						
Medline/PsychINFO (alone)	1.04	1.00	1.00	1.01	1.06	1.27
BIOSIS	1.04	1.00	1.00	1.00	1.06	1.27
CAB Direct	1.04	1.00	1.00	1.01	1.06	1.27
CINAHL	1.04	1.00	1.00	1.01	1.06	1.27
EMBASE	1.04	1.00	1.00	1.00	1.06	1.27
ERIC	1.04	1.00	1.00	1.01	1.06	1.27
HAPI	1.04	1.00	1.00	1.01	1.06	1.27
IPA	1.04	1.00	1.00	1.01	1.06	1.27
LILACS	1.04	1.00	1.00	1.01	1.06	1.27

**Table 8:** Mean (on the log scale) of the ratios of confidence interval widths of Medline/database estimate to reference standard point estimate for dichotomous outcomes

Database	Mean	Minimum	Q1	Median	Q3	Maximum
Acute Respiratory Infections (*n* = 37)						
Medline (alone)	1.12	1.00	1.00	1.00	1.00	3.17
BIOSIS	1.11	1.00	1.00	1.00	1.00	3.17
CAB Direct	1.12	1.00	1.00	1.00	1.00	3.17
CINAHL	1.12	1.00	1.00	1.00	1.00	3.17
EMBASE	1.09	1.00	1.00	1.00	1.00	3.17
ERIC	1.12	1.00	1.00	1.00	1.00	3.17
HAPI	1.12	1.00	1.00	1.00	1.00	3.17
IPA	1.11	1.00	1.00	1.00	1.00	3.17
LILACS	1.12	1.00	1.00	1.00	1.00	3.17
PsycINFO	1.12	1.00	1.00	1.00	1.00	3.17
Infectious Diseases (*n* = 31)						
Medline (alone)	1.10	1.00	1.00	1.00	1.00	2.67
BIOSIS	1.02	1.00	1.00	1.00	1.00	1.25
CAB Direct	1.10	1.00	1.00	1.00	1.00	2.67
CINAHL	1.10	1.00	1.00	1.00	1.00	2.67
EMBASE	1.08	1.00	1.00	1.00	1.00	2.67
ERIC	1.10	1.00	1.00	1.00	1.00	2.67
HAPI	1.10	1.00	1.00	1.00	1.00	2.67
IPA	1.09	1.00	1.00	1.00	1.00	2.67
LILACS	1.10	1.00	1.00	1.00	1.00	2.67
PsycINFO	1.10	1.00	1.00	1.00	1.00	2.67
Developmental, Psychosocial and Learning Problems (*n* = 16)						
Medline/PsychINFO (alone)	1.16	1.00	1.00	1.00	1.20	2.14
BIOSIS	1.16	1.00	1.00	1.00	1.20	2.14
CAB Direct	1.16	1.00	1.00	1.00	1.20	2.14
CINAHL	1.16	1.00	1.00	1.00	1.20	2.14
EMBASE	1.16	1.00	1.00	1.00	1.20	2.14
ERIC	1.13	1.00	1.00	1.00	1.20	2.14
HAPI	1.16	1.00	1.00	1.00	1.20	2.14
IPA	1.16	1.00	1.00	1.00	1.20	2.14
LILACS	1.16	1.00	1.00	1.00	1.20	2.14

**Table 9:** Mean of standardized differences of effect sizes for continuous outcomes

Database	Mean	Minimum	Q1	Median	Q3	Maximum
Acute Respiratory Infections (*n* = 19)						
Medline (alone)	0.01	0	0	0	0.03	0.12
BIOSIS	0.01	0	0	0	0.03	0.04
CAB Direct	0.01	0	0	0	0.03	0.12
CINAHL	0.01	0	0	0	0.03	0.12
EMBASE	0.01	0	0	0	0.03	0.9
ERIC	0.01	0	0	0	0.02	0.04
HAPI	0.01	0	0	0	0.03	0.12
IPA	0.02	0	0	0	0.03	0.12
LILACS	0.01	0	0	0	0.03	0.12
PsycINFO	0.01	0	0	0	0.02	0.04
Infectious Diseases (*n* = 6)						
Medline (alone)	0.0006	0.00	0.00	0.00	0.00	0.003
BIOSIS	0.0004	0.00	0.00	0.00	0.00	0.003
CAB Direct	0.0006	0.00	0.00	0.00	0.00	0.003
CINAHL	0.0005	0.00	0.00	0.00	0.00	0.003
EMBASE	0.0007	0.00	0.00	0.00	0.00	0.004
ERIC	0.0006	0.00	0.00	0.00	0.00	0.003
HAPI	0.0006	0.00	0.00	0.00	0.00	0.003
IPA	0.0006	0.00	0.00	0.00	0.00	0.003
LILACS	0.0006	0.00	0.00	0.00	0.00	0.003
PsycINFO	0.0006	0.00	0.00	0.00	0.00	0.003
Developmental, Psychosocial and Learning Problems (*n* = 19)						
Medline/PsychINFO (alone)	0.01	0.00	0.00	0.00	0.02	0.08
BIOSIS	0.01	0.00	0.00	0.00	0.02	0.08
CAB Direct	0.01	0.00	0.00	0.00	0.02	0.08
CINAHL	0.01	0.00	0.00	0.00	0.02	0.08
EMBASE	0.01	0.00	0.00	0.00	0.02	0.08
ERIC	0.004	0.00	0.00	0.00	0.00	0.02
HAPI	0.01	0.00	0.00	0.00	0.02	0.08
IPA	0.01	0.00	0.00	0.00	0.02	0.08
LILACS	0.01	0.00	0.00	0.00	0.02	0.08

**Table 10:** Mean of the ratios of confidence interval widths of Medline/database estimate to reference standard point estimate for dichotomous outcomes

Database	Mean	Minimum	Q1	Median	Q3	Maximum
Acute Respiratory Infections (*n* = 19)						
Medline (alone)	1.17	1.00	1.00	1.00	1.28	2.22
BIOSIS	1.17	1.00	1.00	1.00	1.28	2.22
CAB Direct	1.17	1.00	1.00	1.00	1.28	2.22
CINAHL	1.17	1.00	1.00	1.00	1.28	2.22
EMBASE	1.15	1.00	1.00	1.00	1.28	2.22
ERIC	1.18	1.00	1.00	1.00	1.28	2.22
HAPI	1.17	1.00	1.00	1.00	1.28	2.22
IPA	1.17	1.00	1.00	1.00	1.28	2.22
LILACS	1.17	1.00	1.00	1.00	1.28	2.22
PsycINFO	1.16	1.00	1.00	1.00	1.28	2.22
Infectious Diseases (*n* = 6)						
Medline (alone)	1.27	1.00	1.00	1.00	1.17	2.46
BIOSIS	1.28	1.00	1.00	1.00	1.17	2.54
CAB Direct	1.27	1.00	1.00	1.00	1.17	2.46
CINAHL	1.27	1.00	1.00	1.00	1.17	2.48
EMBASE	1.26	1.00	1.00	1.00	1.00	2.57
ERIC	1.27	1.00	1.00	1.00	1.17	2.46
HAPI	1.27	1.00	1.00	1.00	1.17	2.46
IPA	1.27	1.00	1.00	1.00	1.17	2.46
LILACS	1.27	1.00	1.00	1.00	1.17	2.46
PsycINFO	1.27	1.00	1.00	1.00	1.17	2.46
Developmental, Psychosocial and Learning Problems (*n* = 19)						
Medline/PsychINFO (alone)	1.11	1.00	1.00	1.00	1.06	2.15
BIOSIS	1.11	1.00	1.00	1.00	1.06	2.15
CAB Direct	1.11	1.00	1.00	1.00	1.06	2.15
CINAHL	1.11	1.00	1.00	1.00	1.06	2.15
EMBASE	1.10	1.00	1.00	1.00	1.06	2.15
ERIC	1.09	1.00	1.00	1.00	1.04	2.15
HAPI	1.11	1.00	1.00	1.00	1.06	2.15
IPA	1.11	1.00	1.00	1.00	1.06	2.15
LILACS	1.11	1.00	1.00	1.00	1.06	2.15

## Additional file

Additional file 1: Description and citations of included systematic reviews. This file includes a description of the included systematic reviews, and the citations for all systematic reviews included in the analysis (57 from Acute Respiratory Infections, 38 from Infectious Diseases, 34 from Developmental Psychosocial Learning Problems). (DOCX 47 kb)

## Abbreviations

ARI: Acute respiratory infections
BIOSIS: BioSciences Information Service of Biological Abstracts
CAB: Commonwealth Agricultural Bureau
CABI: Commonwealth Agricultural Bureau International
CENTRAL: Cochrane Central Register of Controlled Trials
CINAHL: Cumulative Index of Nursing and Allied Health Literature
DARE: Database of Abstracts of Reviews of Effectiveness
DPLP: Developmental Psychosocial Learning Problems
ERIC: Education Resources Information Center
HaPI: Health and Psychosocial Instruments
ID: Infectious diseases
IPA: International Pharmaceutical Abstracts
LILACS: Latin American and Caribbean Health Sciences
SR: Systematic review
